# Multislice computed tomography for comprehensive assessment of the heart in acute chest pain: a case report

**DOI:** 10.1186/1757-1626-2-178

**Published:** 2009-10-31

**Authors:** Andreas Horst Mahnken, Anil Martin Sinha

**Affiliations:** 1 Department of Diagnostic Radiology, RWTH Aachen University, Pauwelsstrasse 30, D-52074 Aachen, Germany; 2 Applied Medical Engineering, RWTH Aachen University, Pauwelsstrasse 20, D-52074 Aachen, Germany; 3 Medical Clinic I, RWTH Aachen University, Pauwelsstrasse 30, D-52074 Aachen, Germany; 4 Department of Cardiology, Coburg Hospital, Ketchendorfer Str. 33, D-96450 Coburg, Germany

## Abstract

**Introduction:**

Over the last decade cardiac computed tomography emerged as a non-invasive imaging modality for the assessment of the heart and the coronary arteries. Only recently its use for patient management in the emergency department was suggested.

**Case Presentation:**

We present an 84-year old male patient with concomitant early in-stent restenosis after coronary artery stent placement, myocardial infarction, left and right ventricular thrombi and aortic valve stenosis. Diagnoses were made on emergency cardiac computed tomography. All findings were confirmed by catheter coronary angiography, echocardiography and cardiac magnetic resonce imaging.

**Conclusion:**

The comprehensive emergency work-up by cardiac computed tomography, illustrates the potential value of cardiac computed tomography in the emergency setting.

## Introduction

Over the last decade cardiac multislice computed tomography (MSCT) emerged as a new tool for assessing the coronary arteries, providing a high negative predictive value to exclude coronary artery disease (CAD). It has also been investigated for the assessment of coronary artery stent patency, coronary artery bypass graft patency, ventricular function and myocardial infarction [1]. Subsequently ECG-gated MSCT was used for the non-invasive assessment of patients suffering from acute chest pain and its efficacy for diagnosing acute coronary syndrome could be shown [2,3]. Only recently its potential to reduce costs in the management of emergency patients has also been proved [4]. However, cardiac CT exceeds the simple assessment of the coronary arteries; it is becoming a tool for the comprehensive diagnostic work-up in the emergency setting.

## Case presentation

**Figure 1:**
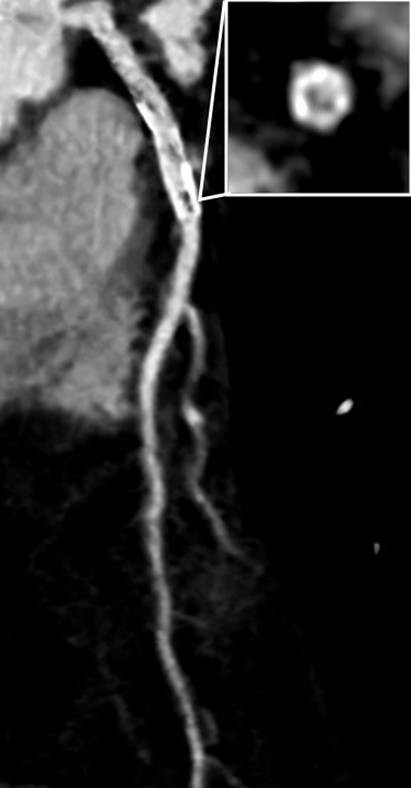
**Curved multiplanar reformat of the LAD shows arteriosclerosis and an eccentric in-stent stenosis that is confirmed by an additional orthogonal view**.

**Figure 2:**
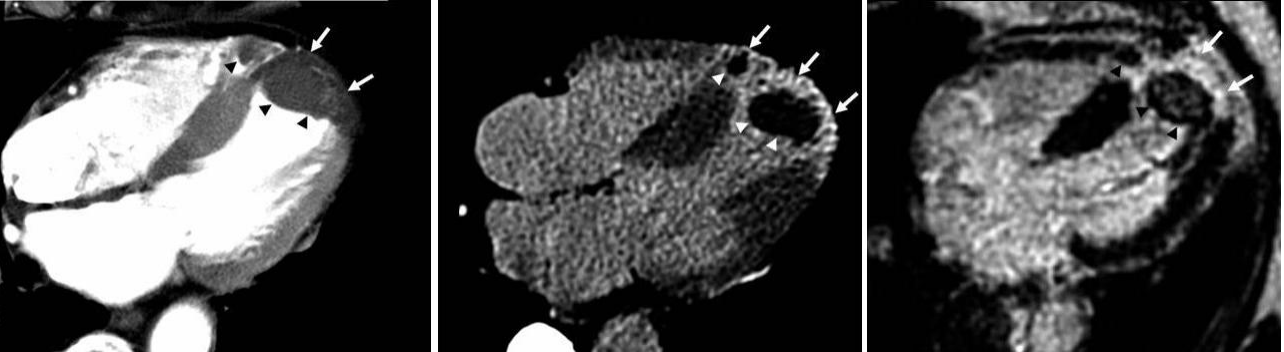
**4-chamber view reformat from arterial phase MSCT reveals a perfusion deficit of the left ventricular apex (A)**. The corresponding late-phase MSCT image presents the delayed myocardial contrast enhancement in the apical-anterior and -septal segments of the left ventricle and the right ventricular apex (B; arrows). Left and right ventricular thrombi are shown (arrowheads). Delayed-enhanced MR image confirms the presence myocardial infarction (arrows) and ventricular thrombi (arrowheads) (C).

**Figure 3:**
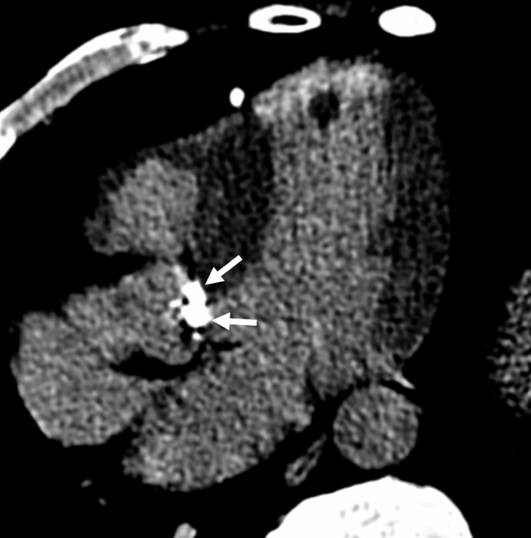
**Aortic valve calcifications (arrows) indicate the presence of aortic valve stenosis**.

One month after an acute ST-elevation myocardial infarction (CK 7230 U/L [<174], CK-MB 557 U/L [<6.7]) with stent placement in the left anterior descending coronary artery, an 84-year-old Caucasian man (180 cm, 75 kg) was admitted to the emergency department with atypical chest pain. Laboratory testing revealed a mild elevation of CK (CK 188 U/L [<174]) and CK-MB (CK-MB 6.9 U/L [<6.7]), while Troponin (T < 0.03 μgL [<0.03]) was negative. His current medication included clopidogrel (75 mg), acetylicsaliciylic acid (100 mg) and (metoprolol 100 mg). The patient's clinical history comprised appendectomy at the age of 16 and a myocardial infarction one month before admission. His cardiovascular risk factors were a history of smoking (45 pack/years) as well as arterial hypertension. The physical examination was unremarkable. There were no new ECG changes comparing to the previous hospital stays. As these findings were considered neither unequivocally indicative for a new occlusive CAD, nor in-stent restenosis, cardiac CT was performed. ECG-gated MSCT coronary angiography (64 × 0.6 mm, 120 kV, 750 mAs_eff._, 80 ml Iopromide 370 injected at 4 ml/s) revealed an in-stent restenosis (Figure 1). An early perfusion deficit was detected during the arterial phase imaging. Late phase images were obtained 10 minutes after contrast injection showing a transmural late-enhancement in the apical-anterior, the apical-septal segments of the left ventricle as well as the right ventricular apex. Moreover, cardiac MSCT depicted left and right ventricular thrombi (Figure 2). Extensive aortic valve calcifications raised the suspicion for aortic valve stenosis (Figure 3).

Based on the CT examination the patient underwent coronary angiography confirming an in-stent restenosis, which was treated with balloon angioplasty. Aortic valve stenosis was echocardiographically confirmed with a mean transvalvular gradient of 24 mm/Hg and MR imaging confirmed the extent of the myocardial late-enhancement and ventricular thrombus. For treating ventricular thrombi the patient was put on warfarin. Enzyme levels returned to normal within 48 hours after angioplasty. Three days later he was discharged in good condition. On follow-up echocardiography 6 months after discharge from hospital the thrombus had completely resolved.

## Discussion

Beside the assessment of the coronary arteries, cardiac MSCT is capable of depicting complex cardiac conditions. In this case, MSCT proved its value for a comprehensive imaging workup in a patient with complex cardiac pathology. MSCT triggered additional diagnostic work-up and therapy for previously unknown left-ventricular thrombus. In comparison with magnetic resonance (MR) imaging, it performed equally well regarding the visualization of the sequels and the detection of complications of myocardial infarction [5]. Although this indication is currently considered uncertain for routine use in the emergency setting [6], cardiac MSCT may be used in well selected emergency patients, providing a comprehensive non-invasive evaluation not only of the coronary arteries, but of the entire heart.

## Abbreviations

CAD: coronary artery disease; CK: creatine kinase; CT: Computed Tomography; ECG: electrocardiogram; LAD: left anterior descending branch of the left coronary artery; MR: magnetic resonance; MSCT: multislice spiral computed tomography; T: Troponin; U: units.

## Competing interests

The authors declare that they have no competing interests.

## Authors' contributions

AHM analyzed and interpreted cardiac CT data and reviewed the relevant literature. AMS was the responsible cardiologist and reviewed the relative literature. All authors have read and approved the final version of the manuscript.

## Consent

Written informed consent was obtained from the patient for publication of this case report and accompanying images. A copy of the written consent is available for review by the journal's Editor-in-Chief.
